# Enhanced Instructed Fear Learning in Delusion-Proneness

**DOI:** 10.3389/fpsyg.2022.786778

**Published:** 2022-04-13

**Authors:** Anaïs Louzolo, Rita Almeida, Marc Guitart-Masip, Malin Björnsdotter, Alexander Lebedev, Martin Ingvar, Andreas Olsson, Predrag Petrovic

**Affiliations:** ^1^Department of Clinical Neuroscience, Karolinska Institutet, Stockholm, Sweden; ^2^Department of Neuroscience, Karolinska Institutet, Stockholm, Sweden; ^3^Department of Neurobiology, Care Science and Society, Karolinska Institutet, Stockholm, Sweden

**Keywords:** delusion-proneness, instructed fear learning, classical fear conditioning, nocebo effect, fMRI, orbitofrontal cortex, expectations, priors

## Abstract

Psychosis is associated with distorted perceptions and deficient bottom-up learning such as classical fear conditioning. This has been interpreted as reflecting imprecise priors in low-level predictive coding systems. Paradoxically, overly strong beliefs, such as overvalued beliefs and delusions, are also present in psychosis-associated states. In line with this, research has suggested that patients with psychosis and associated phenotypes rely more on high-order priors to interpret perceptual input. In this behavioural and fMRI study we studied two types of *fear learning*, i.e., *instructed fear learning* mediated by verbal suggestions about fear contingencies and *classical fear conditioning* mediated by low level associative learning, in delusion proneness—a trait in healthy individuals linked to psychotic disorders. Subjects were shown four faces out of which two were coupled with an aversive stimulation (CS+) while two were not (CS-) in a fear conditioning procedure. Before the conditioning, subjects were informed about the contingencies for two of the faces of each type, while no information was given for the two other faces. We could thereby study the effect of both classical fear conditioning and instructed fear learning. Our main outcome variable was evaluative rating of the faces. Simultaneously, fMRI-measurements were performed to study underlying mechanisms. We postulated that instructed fear learning, measured with evaluative ratings, is stronger in psychosis-related phenotypes, in contrast to classical fear conditioning that has repeatedly been shown to be weaker in these groups. In line with our hypothesis, we observed significantly larger instructed fear learning on a behavioural level in delusion-prone individuals (*n* = 20) compared to non-delusion-prone subjects (*n* = 23; *n* = 20 in fMRI study). Instructed fear learning was associated with a bilateral activation of lateral orbitofrontal cortex that did not differ significantly between groups. However, delusion-prone subjects showed a stronger functional connectivity between right lateral orbitofrontal cortex and regions processing fear and pain. Our results suggest that psychosis-related states are associated with a strong instructed fear learning in addition to previously reported weak classical fear conditioning. Given the similarity between nocebo paradigms and instructed fear learning, our results also have an impact on understanding why nocebo effects differ between individuals.

## Introduction

Clinical observations of patients with psychosis suggest that these individuals have difficulties to focus on one stimulus at a time, especially in an acute psychotic state. Instead, their attention often quickly shifts between different irrelevant stimuli that they perceive as highly salient. The same individual may simultaneously have a set of delusions that are resistant to change, despite being extremely unlikely or even bizarre to most people. The paradox that poorly reliable low-level processes (such as unstable perceptions) co-exist with overly stable high-level beliefs (such as delusions) is of central interest in psychosis research (Sterzer et al., 2008; Moller et al., 2021). Here, we used a task combining instructed fear learning (Mertens et al., 2018) and classical fear conditioning (Fullana et al., 2016) in order to test whether belief formation induced by instructions is stronger in high delusion-proneness—a trait associated with psychotic disorders that is expressed in healthy subjects (van Os et al., 2009)—compared to controls.

Mirroring the clinical picture of unstable perceptions described above, experimental research supports the idea that low-level processes are dysfunctional in schizophrenia and related endophenotypes (Javitt and Freedman, 2015). A consequence of noisy perceptual processes would be a less efficient bottom-up learning. This has been suggested for psychosis-related states in various simple learning paradigms including associative learning (Corlett et al., 2007; Corlett and Fletcher, 2012), reward learning (Murray et al., 2008; Roiser et al., 2009; Schlagenhauf et al., 2014) and classical fear conditioning (Jensen et al., 2008; Holt et al., 2009, 2012; Romaniuk et al., 2010; Balog et al., 2013; Tuominen et al., 2021). These studies on patients and related endophenotypes have often shown both a smaller learning effect of the true association and an increased learning effect of non-existent associations, in line with the aberrant salience hypothesis (Kapur, 2003).

In contrast to bottom-up learning, recent studies suggest that the effect of high-level top-down learning is stronger in patients with psychosis, and in delusion-prone subjects, compared to healthy controls (Schmack et al., 2013; Teufel et al., 2015). Namely, after being presented with explicit and consciously accessed information, these individuals use high-level priors in a top-down fashion more readily than controls, in order to interpret simple perceptual input. Such beliefs may be characterised as overly strong and associated with the predisposition of delusion formation (Schmack et al., 2013).

Recently, theories such as the predictive coding hypothesis of psychosis, have suggested an association between information processing deviations and psychotic symptoms (Fletcher and Frith, 2009; Adams et al., 2013; Sterzer et al., 2018). Despite this, the reason for why psychosis related states are associated with overly strong beliefs and delusions in parallel with a noisy perceptual system, is not fully understood. It has been proposed that the formation of delusions is a secondary consequence of adaption to aberrant low-level signals (Kapur, 2003). Alternatively, it may suggest a strategy of integrating explicit information in a proactive manner to facilitate interpretation of a noisy environment in psychosis-related states.

Here, we tested whether delusion-prone subjects integrate explicit information given in advance, to a higher degree than controls in a *social fear learning task*. We hypothesised that verbal suggestions about the threat value of specific social stimuli, i.e., instructed fear learning, would have stronger effect on affective learning in delusion-prone participants than in controls, in sharp contrast to results from previously performed low-level classical fear conditioning studies on psychosis patients (Jensen et al., 2008; Holt et al., 2009, 2012; Romaniuk et al., 2010; Tuominen et al., 2021), and schizotypal individuals (Balog et al., 2013), which have suggested a weaker learning in psychosis associated phenotypes.

In order to test our hypothesis we showed our subjects four faces out of which two were coupled with aversive electric stimulation (CS+) while two were not (CS-) in a fear conditioning procedure. Ahead of the fear conditioning procedure subjects were informed about the contingencies for two of the faces of each type, while no information was given for the two other faces. We could thereby study the effect of both classical fear conditioning and instructed fear learning. Our main outcome measure consisted of explicit evaluation of the presented faces (Petrovic et al., 2008), and involves, therefore, conscious beliefs about the context. We also measured autonomic responses (i.e., skin conductance response) as an index of learning.

Our study also translates to the nocebo effect, that may be defined as the role of negative expectations from suggestions, associative learning and context in producing an aversive outcome (Barsky et al., 2002; Faasse et al., 2019; Colloca and Barsky, 2020).

It has been suggested that the lateral orbitofrontal cortex (lOfc) is a key structure involved in the processing of higher order expectations that influence emotional processing and experience (Petrovic et al., 2010). In line with this, previous functional imaging studies using tasks related to the present, such as instructed fear learning (Tabbert et al., 2011; Atlas et al., 2016) and nocebo responses (Kong et al., 2008; Asghar et al., 2015; Ellerbrock et al., 2015; Freeman et al., 2015; Schienle et al., 2018), have shown increased activation in lOfc and related regions. Other studies where a change in expectations underlies a change in emotional experience including placebo responses (Petrovic et al., 2002, 2005, 2010; Atlas and Wager, 2014; Wager and Atlas, 2015) and cognitive reappraisal (Eippert et al., 2007; Wager et al., 2008; Kanske et al., 2011; Golkar et al., 2012) have also shown the involvement of lOfc. We therefore hypothesised that (1) the behavioural results would be associated with an larger activation in lOfc for instructed stimuli than for non-instructed stimuli for all subjects, and (2) that this effect would be stronger in high delusion proneness vs. low delusion proneness as well as (3) have a differential interaction with regions involved in pain and fear processing.

## Materials and Methods

### Participants

We screened 925 male individuals aged 18 to 35 years (mean = 24.98 years, *SD* = 0.161) for delusion-proneness using *PDI* (*Peters’ Delusion Inventory*-21 items) (Peters et al., 2004). For each PDI item that is endorsed, three dimensions are rated by the participant on a 5-point Likert scale (1–5) in order to assess the level of conviction, distress, and preoccupation related to the given item (i.e., conviction, distress, and preoccupation scores, respectively). The subjects also completed *ASRS* (*World Health Organization Adult ADHD Self-Report Scale*) (Kessler et al., 2005), and *AQ* (*Autism Spectrum Quotient questionnaire*) (Baron-Cohen et al., 2001) to control for sub-clinical tendencies of ADHD (Attention and Hyperactivity disorder) and ASD (Autism Spectrum disorder) (Louzolo et al., 2017). Participants were recruited through social media and filled in online versions of the questionnaires. It was stressed twice that they had to be healthy and without any psychiatric history. Upon submission of their contact details and after giving their consent, participants received a link to the questionnaires and an automatically generated unique ID-code that they used when filling in the questionnaires.

Based on the questionnaire results we selected 51 right-handed male individuals aged 18–35 years; out of which 26 were in the *low delusion proneness group* (*lDP*; PDI scores ranging from 2 to 6), and 25 in the *high delusion proneness group* (*hDP*; PDI scores ranging from 10 to 17). Due to technical issues during the scanning procedures (movement and technical problems with the stimulation device), 8 participants had to be removed from both behavioural and imaging analyses. A total of 43 participants (*lDP*: *n* = 23, PDI mean = 3.78, *SD* = 1.38, and *hDP*: *n* = 20, PDI mean = 12.85, *SD* = 1.84) thus underwent a successful *instructed fear learning* and *classical fear conditioning* procedure in a 3T GE MR scanner and contributed to the behavioural results. Out of those 43 participants, another 3 were removed from the imaging analyses due to large movement artefacts, resulting in a total of 20 participants in each group contributing to the fMRI results (*lDP*: PDI mean = 3.85 and *SD* = 1.37; *hDP*: PDI mean = 12.85 and *SD* = 1.84). The size of the two groups were comparable to previous fMRI studies on conditioning and psychosis related states (Jensen et al., 2008; Holt et al., 2009, 2012; Romaniuk et al., 2010; Balog et al., 2013).

All participants gave once again their informed consent before the experiment, and were paid 450 SEK for their participation. The study was approved by the regional ethical board of Stockholm.^1^

### Stimuli and Apparatus

In the *classical fear conditioning paradigm*, the unconditioned stimulus (*UCS*) consisted of a mildly aversive electric stimulation. Prior to the start of the experiment a pair of Ag/AgCl electrodes (27 × 36 mm) was attached to participants’ left forearm with electrode gel and used to deliver electrical stimulation. Before lying down in the scanner, participants went through a standard work-up procedure, during which stimulation intensity was gradually increased until participants judged it as unpleasant, but not intolerably painful. Stimulus delivery was controlled by a monopolar DC-pulse electric stimulation (STM200; Biopac Systems Inc., Santa Barbara, United States^2^). Each electrical stimulation lasted for 200 ms, co-terminating the presentation of the reinforced CS+ stimuli. The experiment was presented using Presentation^3^ and was displayed on a screen inside the scanner. Participants controlled the computer cursor through the use of a trackball device.

**FIGURE 1:**
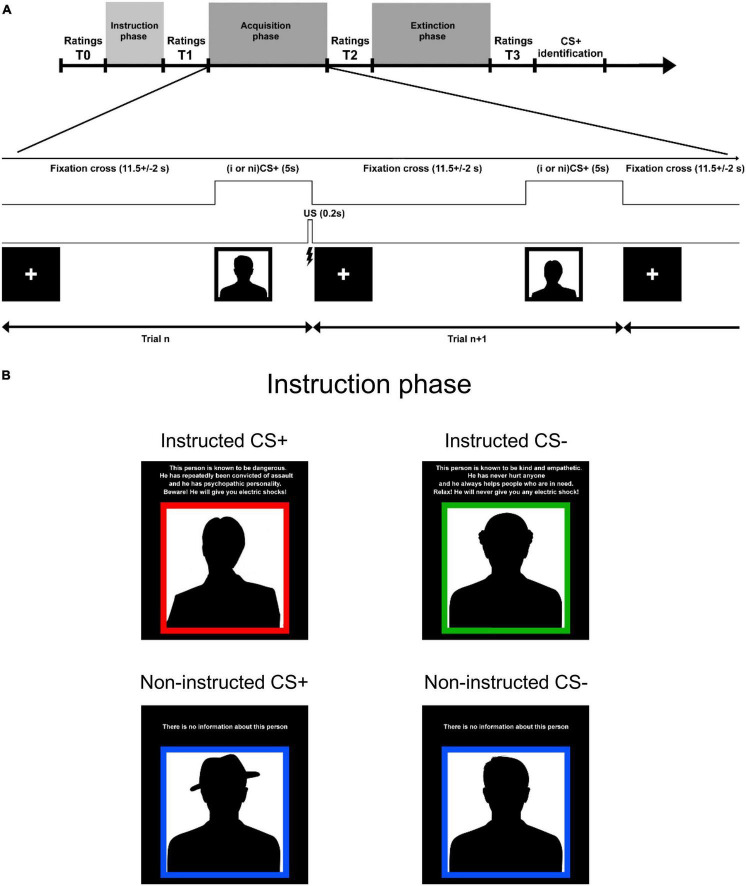
Subjects and experimental design. **(A)** Timeline of paradigm. In the *acquisition and extinction phases* each CS was displayed 12 times for 5 s, and the jittered inter-trial interval was 11.5 ± 2 s. The CS+ were coupled with UCS (mildly painful electric stimulation) with a 50% contingency in the acquisition phase and there was no UCS in the extinction phase. Participants were asked to rate how friendly each CS was experienced, using a visual analogue scale (−100 to 100). In order to estimate learning we calculated the difference between CS- rating and CS+ rating for each CS-pair (instructed and non-instructed). This difference score is referred to as *“affective learning index”’* and the main outcome value in the study. We analysed three *affective learning indices:* (1) T1: after instruction learning, (2) T2: after acquisition, and (3) T3: after extinction. All ratings were normalized in regards to T0. **(B)** In the *instruction phase*, two of the faces (instructed CS+ and CS-; iCS+/iCS-) were coupled with information about their contingencies with the UCS that included a fabricated short description about their personality and the risk of being associated with an aversive stimulation. The two other CS faces (non-instructed CS+ and CS-; niCS+/niCS-) contained no information about their contingencies with the UCS. Instructions were presented twice (followed by ratings–T1’ and T1) in order to increase the effect of information.

The paradigm consisted of a social learning task that started with an *instruction phase* that was followed by a *fear acquisition phase*, and ended with an *extinction phase* (Figure 1A). The conditioned stimuli (CS) consisted of four Caucasian male faces (selected from a picture set used in Johansson et al., 2013) displaying a neutral facial expression (2 CS+ and 2 CS−) and randomised between participants. We used faces, as in several of our previous studies (e.g., Olsson et al., 2005; Petrovic et al., 2008), since they are more salient than abstract figures and we wanted to measure the likability of the different individual faces. Finally, social stimuli also contain more delusion relevant information (as exemplified in paranoia) than many other stimuli. For illustration purposes, we used silhouettes on the timeline sketch Figure 1.

In the instruction phase, two of the faces (instructed CS+ and CS-; *iCS*+/*iCS*-) were coupled with information about their contingencies with the UCS (including a fabricated short description about their personality and the risk of being associated with a “shock”). The two other CS faces (non-instructed CS+ and CS-; niCS+/niCS-) contained no information about their contingencies with the UCS. The phrasing used in the instructions is presented in Figure 1B (original text in Swedish).

In the acquisition and extinction phases each CS was displayed 12 times for 5 s, and the jittered inter-trial interval was 11.5 ± 2 s. The CS+ were coupled with UCS with a 50% contingency in the acquisition phase and there was no UCS in the extinction phase.

### Skin Conductance Response

Skin conductance was recorded during the whole session. Two Ag/AgCl electrodes (27 × 36 mm) were attached to the distal phalange of the first and third fingers of participants’ left hand. The skin conductance response (SCR) was amplified and recorded using an fMRI compatible BIOPAC Systems (Santa Barbara, CA). Data were analysed using AcqKnowledge software (BIOPAC Systems). Processing of the raw data consisted of low-pass (1 Hz) and high-pass (0.05 Hz) filtering. For each CS, the conditioned SCR amplitude was quantified as the peak-to-peak amplitude difference to the largest response, in the 0.5–4.5 s latency window after the stimulus onset. The SCRs were transformed into microSiemens (μS), and responses below 0.02 μS were encoded as zero. A square-root transformation was applied to raw SCRs to normalise the data distribution. Participants who displayed a SCR to less than 20% of each of the two CS+ were considered non-responders and excluded from SCR analyses. This resulted in 18 *lDP* and 20 *hDP* participants that were used in the SCR analysis.

### Behavioural Analyses

Since our focus was on explicit learning we measured *evaluative fear ratings* (Petrovic et al., 2008) for the presented faces. On several occasions throughout the experiment (before instructions, during instructions, before acquisition, before and after extinction) participants had to rate how friendly each CS looked, using a visual analogue scale with “the least sympathetic person you can imagine” stated on the left anchor, and “the most sympathetic person you can imagine” on the right anchor (originally in Swedish). The X-axis coordinates of the scale were converted into numbers, from -100 (left anchor) to +100 (right anchor) and used as the rating scores. The first rating of each CS was referred to as the baseline rating and used to normalise the subsequent ratings for a given CS. The normalised scores were computed for each CS, by subtracting the first ratings from the following ratings. In order to estimate learning in our paradigm we calculated the difference between CS- rating and CS+ rating, in each pair (instructed and non-instructed). This difference score is referred to as “*affective learning index*” and represents the main outcome value in the study as we were interested in explicit learning. Instructions were presented twice (followed by ratings: T1’ and T1) in order to increase explicit learning (Figure 1A). Out of these two ratings we used the one following the second instruction presentation (T1) in subsequent analyses as it represented the total effect of the instruction manipulation. This resulted in three *affective learning indices*: (1) T1-after instruction learning, (2) T2-after acquisition, and (3) T3-after extinction (Figure 1A). During the debriefing session after the experiment, participants were also asked to rate how strongly they felt they had been influenced by instructions and aversive stimulation, respectively (0: no influence at all, 10: extremely high influence).

We used linear mixed models to analyse the effect of the experimental manipulations on the main behavioural outcome variable, i.e., the *affective learning index*. A random effect of subject was modelled, accounting for the repeated measures. The explanatory variables used were subject group (*hDP* vs. *lDP*), the stimulus type (*instructed* vs. *non-instructed*), the phase of the trial (*T1*, *T2*, or *T3*) and the interactions between these variables. Analysis were conducted using the software R 3.2.3 (R Core Team, 2015) using packages lme4 (Bates et al., 2015) and lmerTest (Kuznetsova et al., 2017).

Two specific hypotheses were tested for the behavioural part of the study:

**-*Main hypothesis*:** As psychosis proneness has been associated with stronger higher order learning and use of high-level priors (Schmack et al., 2013; Teufel et al., 2015), instructions should have a greater influence on fear learning in the delusion-prone subjects than in the normal population. We therefore hypothesised that *hDP* would show larger instructed *affective learning index* in all phases compared to *lDP*.

**-*Secondary hypothesis*:** In line with previous studies on classical fear conditioning (Jensen et al., 2008; Holt et al., 2009, 2012; Romaniuk et al., 2010; Balog et al., 2013; Tuominen et al., 2021) we hypothesised that delusion-prone individuals would display an attenuated fear learning. This would be reflected by significantly smaller non-instructed *affective learning index* following acquisition in *hDP* as compared to *lDP*.

In summary, on a behavioural level we expected increased effect of instructions on fear learning (instructed fear learning) but decreased effects of classical fear conditioning related to delusion proneness.

### Functional Imaging Analysis

We hypothesised that lateral orbitofrontal cortex (lOfc) would have a decisive role in the increase of fear learning due to instructions—based on its previously shown involvement in processes where expectations have been experimentally manipulated including instructed fear learning (Tabbert et al., 2011; Atlas et al., 2016), nocebo responses (Kong et al., 2008; Asghar et al., 2015; Ellerbrock et al., 2015; Freeman et al., 2015; Schienle et al., 2018), placebo responses (Petrovic et al., 2002, 2005, 2010; Atlas and Wager, 2014; Wager and Atlas, 2015) and cognitive reappraisal (Eippert et al., 2007; Wager et al., 2008; Kanske et al., 2011; Golkar et al., 2012). Data from these studies suggests that the right lOfc, especially, is involved in placebo (Petrovic et al., 2002, 2005, 2010) and cognitive reappraisal processes (Wager et al., 2008). We therefore examined the acquisition phase results with a primary focus on effects in lOfc. Further, we posited that any behavioural effects in relation to instructed fear learning would be linked to functional or effective connectivity effects in the right lOfc as previously observed in cognitive reappraisal (Wager et al., 2008).

Apart from the general hypothesis about the involvement of lOfc in instruction effects, we more specifically hypothesised that *hDP* (compared to *lDP*) would exhibit (i) increased lOfc responses to instructed fear learning, and (ii) increased effective connectivity between the lOfc, and pain and fear regions, as an underlying mechanism associated with a stronger effect of instructions on *affective learning index*.

Due to limited space, we constrained the present functional imaging analysis to the acquisition phase.

### Image Acquisition

Participants were scanned in a 3T MR General Electric scanner with a 32-channel head coil. A T1-weighted structural image was acquired before the beginning of the paradigm. Functional scans were obtained using a gradient echo sequence T2*-weighted echo-planar imaging (EPI) scan [*TR* = 2.334 s, *TE* = 30 ms, flip angle = 90 degrees, 49 axial slices in ascending order (thickness = 3 mm) and a field of view (FOV) = 22 cm, matrix size = 72 × 72 × 3 mm]. The first four scans were defined as dummy scans and discarded from the analysis. Functional image acquisition comprised 2 runs of 245 volumes each (acquisition and extinction phases, respectively), with a break of approximately 4–5 min between them.

### Imaging Data Analysis

Data pre-processing and analyses were performed using a default strategy in the SPM8 software package (Statistical parametric mapping, Welcome Department of Cognitive Neurology, London, United Kingdom^4^). For each participant, individual images were first slice-time corrected and realigned to the first volume to correct for head movement. The T1-weighted image was then co-registered with the mean EPI image, segmented and normalised to the Montréal Neurological Institute standard brain (MNI). Then, functional images were spatially smoothed with an 8-mm full width at half maximum (FWHM) isotropic Gaussian kernel, and a temporal high-pass filter with a cut-off of 128 s was used to remove low-frequency drifts.

All analysis in the present study focused on the acquisition phase. A general linear model (GLM) comprising nine regressors was defined at the first-level analysis; one regressor per CS type (iCS+, iCS−, niCS+, and niCS−) with each onset modelled as a 5-s event, and one regressor for the UCS presentation. In addition, these four regressors (excluding UCS) were also parametrically modulated with a linearly changing function to capture activity changes over time. All nine regressors were convolved with the canonical haemodynamic response function and entered into the GLM as implemented in SPM. Motion regressors were also included in the model. The two phases of the experiment (acquisition and extinction) were modelled and analysed separately.

We first analysed main effects of fear (CS+ vs. CS−). Similarly, we examined the main effects of pain. We also analysed possible differences between *hDP* and *lDP* in a 2nd level analysis of these activations using a ROI approach in order to increase the sensitivity. A small volume correction in a spherical ROI (6 mm radius) was then applied in the contrasts between the two groups. The ROIs were centred over the maximally activated voxels in caudal ACC (cACC) and anterior insula in the main effect of fear and in posterior insula in the main effect of pain. The results were assessed at *p* < 0.05, family-wise error (FWE) corrected for multiple comparisons.

To test our main hypotheses regarding the functional imaging results, we first conducted a GLM group analysis to compare the effect of instruction in the lOfc for *hDP* to *lDP* participants. The results were assessed at *p* < 0.05, family-wise error (FWE) corrected for multiple comparisons. Given our *a priori* hypothesis, we used small-volume correction (SVC) for multiple comparisons within an anatomical lOfc ROI defined using the pick atlas in the SPM, in addition to an exploratory whole brain analysis.

We also examined effective connectivity using a psychophysiological interaction (PPI) analysis in SPM (Friston et al., 1997). This analysis identifies context-induced changes in the strength of connectivity between brain regions, as measured by a change in the magnitude of the linear regression slope that relates their underlying neuronal responses. Significant PPI results indicate that the contribution of one area to another changes with the experimental context (Friston et al., 1997). We assessed connectivity changes between the right lOfc and the rest of the brain. The lOfc seed region was defined using a sphere with a radius of 6 mm centred on the right lOfc group maximum from the GLM analyses of instruction-related activity. For each participant, the seed was adjusted to centre on the individual peak response within the group seed sphere, and the fMRI time series was extracted and deconvolved to generate the neuronal signal. We then conducted two PPI analyses using the contrast (i) instructed vs. non-instructed [(iCS+ and iCS-) vs. (niCS+ and niCS-)] and (ii) the interaction effect (fear learning in instructed vs. fear learning in non-instructed stimuli; [(iCS+ vs. iCS-) vs. (niCS+ vs. niCS-)]) as the psychological factor. For each participant, a GLM was conducted including three regressors representing the time course of the seed region (the physiological factor), the psychological factor and their product (the PPI). The parameter estimates for the PPI regressor from each participant were then entered into a second-level analysis, and we again assessed the results at pFWE < 0.05.

We conducted SVC in several ROIs for the PPI analyses. First, we used the group-level main effect of fear learning (CS+ vs. CS-) to identify cACC and anterior insula (Supplementary Table 1). Second, we examined any group differences in low-level sensory processing areas, in line with previous findings of altered effective connectivity between the lOfc and the visual cortex in a visual expectation manipulation task related to delusion proneness (Schmack et al., 2013). To obtain a low-level sensory region, we used the group-level main effect pain (mildly painful electric stimulation) to identify the posterior insular cortex. This region has been the most consistently reported brain activation site across all pain conditions and is considered a nociceptive input area (Tanasescu et al., 2016).

Finally, we assessed whether there was a significant correlation between conviction scores and the functional connectivity between the lOfc seed-region and low-level sensory regions (i.e., defined as posterior insular in the present study) to investigate whether we could reproduce the findings by Schmack et al. (2013). On a more exploratory level, we analysed whether such a correlation was also present for the total PDI-score, the normalised conviction score as well as the two other sub-scores in PDI (distress score and preoccupation scores).

## Results

In the present study, we show behavioural results that either involve all phases together or the instruction and acquisition phase separately as well as the fMRI-results from the acquisition phase in order to study our predefined hypotheses. The study results have previously been presented in bioRxiv (Louzolo et al., 2019). Behavioural and fMRI results specifically focusing on extinction phase will be presented elsewhere.

## Behavioural Results

### Ratings

#### Baseline Ratings

A baseline rating (T0) was collected for each face before any information was presented and it was used for normalisation of subsequent ratings (Figure 1A). We tested whether groups (*hDP* and *lDP*) differed on the averaged absolute value of the initial baseline ratings, and found no significant difference (*t* = 0.092, *p* = 0.927, independent two-sample *t*-test). This result suggests that any possible group differences associated to instructions or conditioning cannot be explained simply by a difference between the groups in their general rating strategy.

#### Affective Learning Index

The main behavioural outcome measure of the study was the *affective learning index*, which reflects how subjects change the ratings given to CS- vs. CS+ stimuli after conditioning or instructions.

As a general control of the paradigm, effects of instructed fear learning and classical fear condition were first analysed independently in the two groups (lDP and hDP). Evaluative fear learning measured with *affective learning index* was observed after instructions (T1 vs. T0) for instructed stimuli and after acquisition phase (T2 vs. T1) for both instructed (threshold level) and non-instructed stimuli independently for both lDP and hDP. Thus, learning as a consequence of instructed fear learning and classical fear conditioning were accomplished for both groups independently.

A mixed linear model was used to study the effects of subject group (*hDP* vs. *lDP*), stimulus type (*instructed* vs. *non-instructed*), phase of the trial (*T1*, *T2*, or *T3*) and the interactions between these variables on the *affective learning index*. We found *significant effects of group* (*p* = 0.029), *stimulus type* (*p* < 0.00001), and *phase* (*p* < 0.00001). The three-way interaction between these variables as well as the interaction between group and phase were not significant (*p* = 0.750 and *p* = 0.167, respectively). However, there was an *almost significant stimulus type × group interaction* (*p* = 0.057) and a *significant stimulus type × phase interaction* (*p* = 0.00003). This means that the effect of instructions depends on the group (according to our main hypothesis) and also on the phase. Since there were interaction effects with the stimulus type, in order to study the effects of group and phase, we divided the data into two sets, corresponding to the instructed and non-instructed stimuli.

**FIGURE 2:**
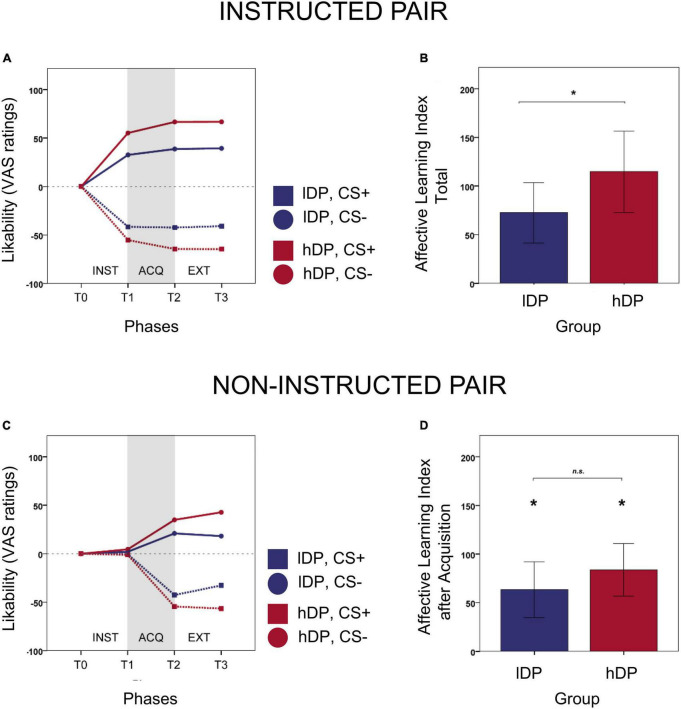
Behavioural results of instructed and non-instructed learning. **(A)** Timeline of likability ratings for instructed CS-stimuli over the three phases. **(B)** In line with our hypothesis the *affective learning index* was higher for the *hDP* (mean = 125.77, *SD* = 93.06) than for the *lDP* (mean = 74.50, *SD* = 67.98) for the instructed CS-stimuli and we found a significant group effect (*p* = 0.044) over all phases thus confirming our main hypothesis. **(C)** Timeline of likability ratings for non-instructed CS-stimuli over the three phrases. **(D)** For the non-instructed stimuli, there was no significant effect of group (*p* = 0.105). The average non-instructed *affective learning index* after acquisition was somewhat larger (albeit non-significant) in the delusion-prone group than in the control group (*hDP*: mean = 89.45, *SD* = 81.52; *lDP*: mean = 63.00, *SD* = 62.16). As suggested from the time-line the largest part of this non-significant difference was dependent on T3 (ratings after extinction). At phases T2 the affective learning index was significantly larger than 0 (*p* < 0.00001) for each group, indicating that the fear conditioning worked and the subjects learned the contingencies. Error bars: 2 S.E.

For the *instructed stimuli* (Figures 2A,B), there was a significant effect of group (*p* = 0.044), but not of phase (*p* = 0.109). The *affective learning index* was higher for the *hDP* (mean = 125.77, *SD* = 93.06) than for the *lDP* (mean = 74.50, *SD* = 67.98) thus confirming our main hypothesis. We also extended the model to include the interaction between group and phase. The interaction was not significant (*p* = 0.26), indicating that the group effect is present in all phases. The *affective learning index* was significantly larger than zero for *lDP* (*p* = 0.0002). Thus, for the instructed stimuli, the *affective learning index* was larger than zero for all groups and phases, confirming that there was an effect of instructions in both groups, that persisted for all phases.

For the *non-instructed stimuli* (Figures 2C,D), there was a significant effect of phase (*p* < 0.00001), but not of group (*p* = 0.105). The *affective learning index* was not different from 0 at phase T1. This is expected since, for non-instructed stimuli, at T1 the subjects had no more information than at T0. At phases T2 and T3 the *affective learning index* was significantly larger than 0 (*p* < 0.00001), indicating that the classical fear conditioning worked and the subjects learned the contingencies.

To test the *secondary hypothesis*, the model on the non-instructed stimuli was extended to include the interaction between group and phase. The interaction was almost significant (*p* = 0.056). Hence, to be able to interpret the effects of group, we analysed the data for each phase separately. However, there was no significant effect of group for T1 and T2 (*p* = 0.653 and *p* = 0.235, respectively) and only an effect for T3 (*p* = 0.025). Namely, after the acquisition phase (T2) for the non-instructed stimuli there was no difference in *affective learning index* between the two groups of subjects. The effect of extinction (associated with ratings at T3) is further elaborated elsewhere.

### Skin Conductance

A one-tailed *t*-test on the differential SCR (SCR-CS+ vs. SCR-CS-) in the acquisition phase for all subjects together, was significantly different from zero (mean = 0.0151, *SD* = 0.0271; *t* = 3.424, *df* = 37, *p* = 0.001 one-tailed) suggesting a significant conditioning. This was also the case for each group, when analysed separately (*lDP* mean = 0.0126 μS, *SD* = 0.0248, one-sample *t*-test *t* = 2.145, *df* = 17, *p* = 0.024 one-tailed—*hDP* mean = 0.0174 μS, *SD* = 0.0296, one-sample *t*-test *t* = 2.628, *df* = 19, *p* = 0.009 one-tailed). There was no group difference (independent two-sample *t*-test *t* = −0.741, *df* = 73, *p* = 0.461).

The differential SCR was mainly driven by the iCS-pair as suggested by a significant difference between the instructed and non-instructed condition in *lDP* (instructed mean = 0.0266 μS, *SD* = 0.036, non-instructed mean = −0.015 μS, *SD* = 0.029; paired *t*-test *t* = 2.780, *df* = 17, *p* = 0.014) and in *hDP* (instructed mean = 0.0251 μS, *SD* = 0.031, non-instructed mean = 0.010 μS, *SD* = 0.036; paired *t*-test *t* = 2.188, *df* = 19, *p* = 0.042). However, there was no significant interaction between the groups (*hDP* or *lDP*) and condition (instructed or non-instructed).

Overall, it should be noted that the SCR data recorded in the fMRI scanner was noisy. We only used participants who showed a SCR to at least 20% of the presentations of each CS (hence, considered as responders; *n* = 38). However, many of them were characterised by a low reactivity.

### Effects of Peters’ Delusion Inventory Sub-Scores on Ratings

**FIGURE 3:**
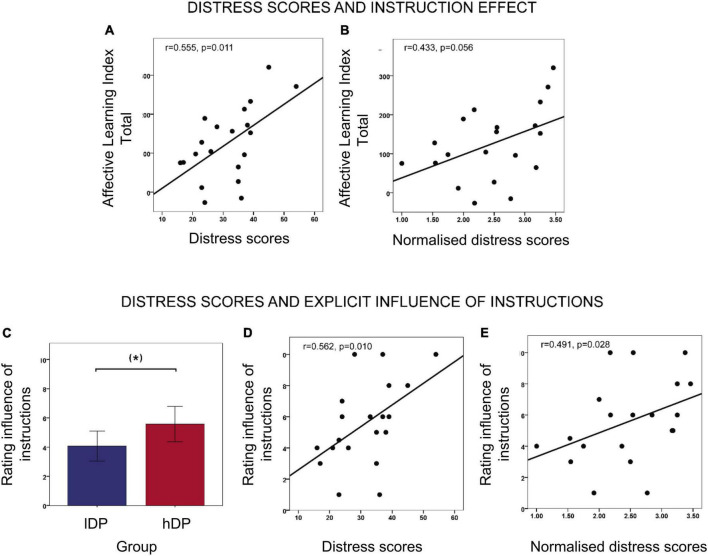
Relation between instruction effects and delusional distress. **(A)** Correlations between *distress scores* and overall instructed *affective learning index* (averaged over three phases) in *hDP* (*r* = 0.555, *p* = 0.011, Pearson correlation tests). **(B)** Correlations between normalised *distress scores* and overall instructed *affective learning index* (averaged over three phases) in *hDP* (*r* = 0.433, *p* = 0.056, Pearson correlation tests). **(C)** Rating of the explicit influence of instructions in *lDP* and *hDP*. The group difference is on the border of significance *t* = −1.910, *p* = 0.063, *df* = 40 (independent two-sample *t*-test). **(D)** Correlation between distress scores and explicit rating of instruction influence in *hDP* (*r* = 0.562, *p* = 0.010, Pearson correlation tests). **(E)** Correlation between normalised distress scores and explicit rating of instruction influence in in *hDP* (*r* = 0.491, *p* = 0.028, Pearson correlation tests). Error bars: 2 S.E.

In an exploratory analysis, we investigated whether PDI scores and their components (distress, preoccupation and conviction) were related to the different ratings for instructed stimuli in *lDP* and *hDP*, respectively. In *hDP* we observed a significant correlation between distress scores and the overall instructed *affective learning index* (*r* = 0.555, *p* = 0.011 Pearson correlation tests) (Figure 3A), as well as the instructed *affective learning index* in T1 (after instructions; *r* = 0.614, *p* = 0.004) and T2 (after acquisition; *r* = 0.518, *p* = 0.019). While similar correlations were observed for preoccupation and conviction scores, they did not reach significance. No significant correlations between distress scores and a*ffective learning index* were found for *lDP*.

Since distress seemed to be an important variable in relation to effects of instructions in our fear learning paradigm, we explored it further. Only analysing the total sum of each of these sub-scores without controlling for the Yes/No score can be somewhat misleading, as it makes it difficult to differentiate between people who would score high on distress because they have a few delusion-like experiences that are extremely distressing, from people who score as high on distress because they have many delusion-like experiences that are not distressing at all. Normalising to the number of endorsed items (number of “yes” answers, or the so-called “total PDI score”) provides a better estimate of how distressed participants are, unrelated to whether there is one or several delusion-like experiences. We therefore also compared *the two groups* in terms of normalised sub-scores and found that the average normalised distress score in *hDP* was significantly larger than in *lDP* (*hDP* = 2.47, *lDP* = 1.95; independent sample *t*-test *t* = −2.593, *p* = 0.013, *df* = 41). Moreover, in *hDP*, the normalised distress score also correlated positively with *affective learning index* after the instruction phases (*r* = 0.527, *p* = 0.017, Pearson correlation tests) (Figure 3B). This correlation only reached a trend level after the acquisition phase (T2), as well as when considering the three phases together (*r* = 0.400, *p* = 0.080; *r* = 338, *p* = 0.091, respectively—Pearson correlation tests). No significant correlations between normalised distress scores and a*ffective learning index* were found for *lDP*.

#### Post-experiment Ratings

After the experiment, participants were asked to explicitly rate the influence of instructions, and pain stimuli (respectively) from 0 to 10. An independent sample *t*-test revealed a trend towards a larger influence of instructions reported in the *hDP*, compared to the *lDP* (mean *lDP* = 4.07, *SD* = 2.42, mean *hDP* = 5.58, *SD* = 2.69; *t* = −1.910, *p* = 0.063, *df* = 40 two-tailed) (Figure 3C), while there was no group difference in terms of pain influence.

Interestingly, in the delusion-prone group the explicit rating of instruction influence was also significantly correlated to the distress sub-score (*r* = 0.562, *p* = 0.01 Pearson correlation tests) (Figure 3D) and with the normalised distress score (*r* = 0.491, *p* = 0.028 Pearson correlation tests) (Figure 3E).

## Functional Imaging Results

**FIGURE 4:**
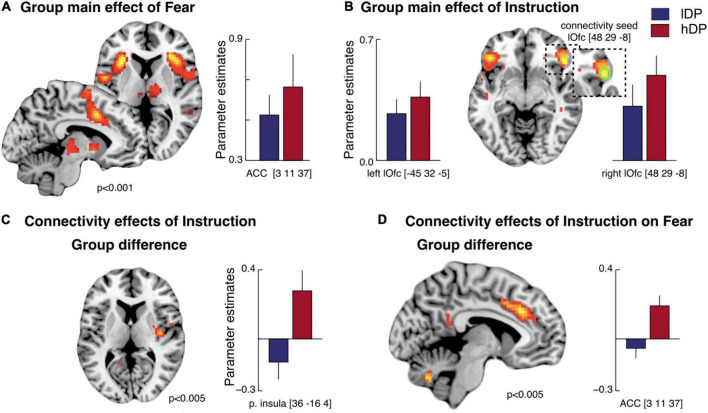
Brain activity related to the effects of conditioning and instructions—BOLD response **(A,B)** and PPI analyses **(C,D)**. **(A)** Main effect of fear (*CS*+ *vs. CS*-): an activation in caudal anterior cingulate cortex (cACC), bilateral anterior insula, premotor/dorsolateral prefrontal cortex (dlPFC), right temporo-parietal junction (rTPJ) was observed (Supplementary Table 1). The activation pattern was similar for instructed (iCS+ vs. iCS-) and non-instructed (niCS+ vs. niCS-) stimuli. No group difference was observed. **(B)** Main effect of instructions: bilateral activations in lateral orbitofrontal cortex (lOfc) (ROI analysis and whole brain analysis) and an activation in dlPFC (whole brain analysis) were observed (Supplementary Table 2). This effect was mainly driven by the *hDP*, and only this group showed significant activations in lOfc (bilateraly). **(C)** A psychophysiological interaction (PPI) analysis on the effect of instructions (vs. non-instructed trials): An increased connectivity between the right lOfc and functionally defined low-level pain processing areas (i.e., right posterior insula) (*Z* = 3.29, pFWE = 0.004) was observed specifically in *hDP* (i.e., compared to in *lDP*). **(D)** A PPI-analysis on the effects of instruction on fear processing: A larger connectivity between lOfc and the cACC (overlapping with fear related activation) was observed in *hDP* than in *lDP* (*Z* = 2.96, pFWE = 0.012). Error bars: S.E.

A simultaneous fMRI measurement showed that the main effect of conditioning (i.e., all CS+ vs. all CS- in the acquisition phase) led to activations in brain areas that are consistently reported in studies of classical fear conditioning (Fullana et al., 2016). These included anterior insula, caudal anterior cingulate cortex and thalamus bilaterally as well as brainstem (Figure 4A and Supplementary Table 1). However, no significant differences were observed between the groups in the regions of interest (ROI) analysis for (CS+ vs. CS-).

In line with our hypothesis, we observed a main effect of instructions [(iCS+ + iCS-) vs. (niCS+ + niCS-)] in lateral orbitofrontal cortex (lOfc) for all subjects (Figure 4B and Supplementary Table 2)—driven mainly by *hDP* subjects (only this group showed significant activations in lOfc; Supplementary Table 2). This suggests a plausible underlying prefrontal mechanism associated with the observed behavioural effects of instructions on fear learning. In addition, *hDP* individuals also displayed activation in the ventromedial prefrontal cortex (vmPFC) that was not observed in the *lDP*, nor in the all-subject activations (Supplementary Table 2). However, there were no significant differences between the groups in the main effects of instructions (subtraction analysis).

For completeness, we analysed the effect of fear learning specifically for the instructed (Supplementary Table 3) and non-instructed stimuli (Supplementary Table 4). These analyses overall resembled the main effect of conditioning and did not reveal and significant differences between *hDP* and *lDP*. Our final contrast analysis focused on the main effect of pain for all subjects, and showed activations in region previously implicated in pain processing including bilateral insula and cACC (Supplementary Table 5).

**FIGURE 5:**
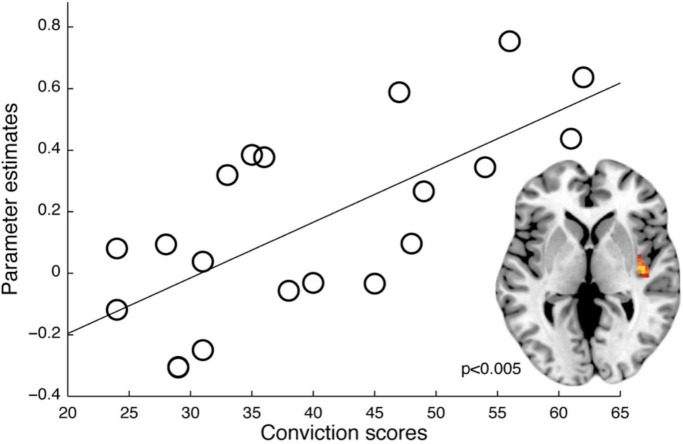
Relation between delusion-proneness and functional connectivity. The functional connectivity (PPI-analysis) between the right lOfc and i.e., right posterior insula ROI as an effect of instructions (vs. non-instructed trials) correlated with conviction scores in the *hDP* (*Z* = 3.44, pFWE = 0.003). A similar effect was shown for PDI-total scores (*Z* = 3.29, pFWE = 0.004) and normalised conviction scores also in the *hDP* (*Z* = 2.77, pFWE = 0.016).

A psychophysiological interaction (PPI) analysis revealed increased connectivity in instructed trials (vs. non-instructed trials) specifically for *hDP* (i.e., compared to *lDP*) between the right lOfc and functionally defined nociceptive input region (right posterior insula) (*Z* = 3.29, corrected *p* = 0.004), supporting previous findings of an association between sensory processing and lOfc in delusion-prone individuals (Schmack et al., 2013; Figure 4C). Moreover, PPI-analysis of the effects of instruction on fear processing showed a significantly larger connectivity between the lOfc and the caudal anterior cingulate cortex (cACC), overlapping with fear related activation, in *hDP* compared to *lDP* (*Z* = 2.96, corrected *p* = 0.012) (Figure 4D). Last, we tested whether we could conceptually replicate the correlation reported in earlier work, between conviction scores and functional connectivity in instructed trials between the right lOfc and functionally defined early sensory processing regions (Schmack et al., 2013) (i.e., right posterior insula, here), specifically for *hDP* individuals (i.e., compared to *lDP*). This analysis showed a significant effect (pFWE = 0.003) (Figure 5), that was also observed when the PPI-analysis was correlated with the total PDI score (pFWE = 0.004) and the normalised convictions scores (pFWE = 0.016).

## Discussion

The present findings confirmed our main hypothesis stating that the effect of instructions on fear learning, i.e., instructed fear learning, would be larger in the delusion-prone group (*hDP*) than in the control group (*lDP*) (Figures 2A,B). The effect was shown in the affective learning index for the instructed stimuli, where evaluative ratings of instructed CS+ faces were compared to instructed CS- faces. However, we did not observe any significant group difference in non-instructed fear learning (classical fear conditioning) (Figures 2C,D). Our results mirror recent studies reporting an increased effect of high-level priors on perceptions in psychosis-related states (Schmack et al., 2013; Teufel et al., 2015) and extend these observations to instructed fear learning. Importantly, as we measured evaluative social ratings as our outcome variable, we also targeted the participants’ specific beliefs about different social stimuli. Thus, in contrast to the aforementioned studies (Schmack et al., 2013; Teufel et al., 2015), we argue that in psychosis-related states, explicit beliefs about the world are also more susceptible to be changed after explicit learning. In addition, our data suggests that *hDP* individuals displayed a larger *affective learning* than *lDP* individuals after instructions, already before the CS-UCS pairing. In other words, they had already formed stronger beliefs that biased their experience of the faces, even before low-level learning in the acquisition phase. Thus, we expand previous views on delusion formation as a secondary mechanism in which the individual tries to explain specific aberrant stimuli (Kapur, 2003), by suggesting that formation of such beliefs might also represent a pro-active coping strategy in order to facilitate interpretation of an unstable environment.

Instructed fear learning (Mertens et al., 2018) has many similarities to nocebo treatment effects (Barsky et al., 2002; Faasse et al., 2019; Colloca and Barsky, 2020), in that both often involve a suggestion that an experience will be unpleasant or aversive. More specifically, in instructed fear learning the subject is informed that a specific event (Stimulus 1) is associated with and predicts an aversive stimulus (Stimulus 2). The effects on subsequently shown Stimuli 1 are then measured in ratings, autonomic measures or brain responses. In nocebo paradigms, the subject is typically informed that a treatment or an event (Stimulus 1) is associated with an increased unpleasant or aversive experience induced by an aversive stimulus such as a painful event (Stimulus 2). The nocebo effect is measured when Stimulus 2 is presented using ratings, autonomic measures or brain responses. Thus, while instructed fear learning is focused on the anticipation phase of an unpleasant event, the nocebo effect is focused on the unpleasant event itself. Also, while instructed fear learning just informs the subject about a relation, the nocebo paradigm gives suggestion about the nature of a stimulus. Both instructed fear learning and nocebo paradigms may also involve a conditioning procedure, but verbal suggestions are of key importance in the experimental paradigms (Mertens et al., 2018; Colloca and Barsky, 2020). In fact, nocebo studies suggest that verbal suggestions may fully mediate the effect, in contrast to placebo studies where the conditioning has additive effects (Colloca et al., 2008). Similarly, instructions mediate a strong effect on fear learning (Mertens et al., 2018) that cannot be completely overridden by subsequent situational safety information (Mertens et al., 2016). Given the similarities between instructed fear learning and nocebo effects, our results suggest that high delusion proneness may be associated with stronger explicit nocebo-like effects than low delusion proneness.

In the present study, we focused on delusion proneness, a personality trait in healthy individuals that includes subclinical levels of delusional ideation (Peters et al., 2004; van Os et al., 2009). Cognitive, thought- and perceptual mechanisms underlying delusion- and psychosis-proneness are considered to be similar to the one underlying psychosis (Peters et al., 2004; van Os et al., 2009; Fusar-Poli et al., 2013; Teufel et al., 2015). As this phenotype is dimensionally expressed in humans, all individuals are more or less prone to this type of behaviour and related information processing. Thus, this trait has significant impact on variability in human behaviour among healthy subjects. However, we propose that similar effects of top-down high-level learning may be present in psychosis patients.

The effect of instructions on fear learning was also significantly related to the degree of *delusional distress* in the *hDP*. This finding was still present when distress scores were normalised, such that they did not depend on the number of endorsed delusional items, which underscores the importance of this dimension in belief formation. These findings may be of special interest since it has been suggested that psychosis-related states characterised with more distress and help seeking are also associated with a larger risk to convert into a clinical psychotic disorder (Fusar-Poli et al., 2013).

We failed to show that *hDP* was associate with lower classical fear conditioning than *lDP* for the non-instructed condition as initially hypothesised. In fact, the average non-instructed *affective learning index* after acquisition (i.e., evaluative ratings) was somewhat larger, albeit non-significant, in *hDP* compared to *lDP* (Figure 2D). At first glance, this result seems to contrast with previous studies showing a smaller classical fear conditioning effect in psychosis patients (Jensen et al., 2008; Holt et al., 2009, 2012; Romaniuk et al., 2010; Balog et al., 2013; Tuominen et al., 2021) and schizotypal individuals (Balog et al., 2013) suggestive of a weaker bottom-up learning in these phenotypes. However, it is important to keep in mind that our non-instructed condition may involve a faster development of explicit beliefs about contingencies compared to ordinary classical fear conditioning experiments, due to the presence of an instructed condition in the same experiment. Thus, our non-instructed fear learning cannot be simply compared to standard classical fear conditioning studies. Future studies will have to control for such confounding effects when comparing instructed vs. non-instructed conditions.

Apart from the effects of fear learning measured with *affective learning index*, the subjects also explicitly rated how much the painful stimulation and the instructions affected them. Interestingly, although no group difference was observed for the painful stimulation, the *hDP* tended to rate that they were more affected by the instructions than the *lDP*. Also, this effect was significantly correlated with the delusional distress for the instructed stimuli in the *hDP* (similarly to the *affective learning index*). Thus, subjects in the *hDP* group seem to have a metacognitive awareness of the fact they are highly affected by explicit information.

Our fMRI results revealed that the main effect of conditioning led to activations in brain areas that are consistently reported in classical fear conditioning studies including caudal ACC, anterior insula, thalamus and brainstem (Fullana et al., 2016), but no group differences were reported (Figure 4A and Supplementary Table 1).

In line with our hypothesis, we observed a main effect of instructions in lateral orbitofrontal cortex (lOfc) for all subjects (Figure 4B and Supplementary Table 2)—driven mainly by *hDP* as only this group showed a significant (and bilateral) activation in lOfc. Increased activation in the orbitofrontal cortex has previously been shown in imaging studies involving both instructed fear learning (Tabbert et al., 2011; Atlas et al., 2016) and nocebo effect (Kong et al., 2008; Asghar et al., 2015; Ellerbrock et al., 2015; Freeman et al., 2015; Schienle et al., 2018) as well as in placebo treatment studies (Petrovic et al., 2002, 2005, 2010; Atlas and Wager, 2014; Wager and Atlas, 2015) and cognitive reappraisal (Eippert et al., 2007; Wager et al., 2008; Kanske et al., 2011; Golkar et al., 2012). All these experimental paradigms involve an explicit change in the underlying rules relating to how to interpret an emotional experience and the associated expectations. Also, the activity seems to be independent of expected value. In a predictive coding framework, which has previously been applied to the placebo effect (Petrovic et al., 2010; Buchel et al., 2014), the lOfc may thus be a key region for higher order priors. A related research line suggests that the orbitofrontal cortex is important for learning task-state representations, especially when hidden information is important for the task (Niv, 2019). This may be compared to the presented paradigms above, that contained hidden information about how a stimuli should be interpreted, given in the instruction phase. This suggests a plausible underlying prefrontal mechanism associated with the observed behavioural effects of instructions on fear learning—an effect that was significantly larger in the *hDP* than in the *lDP*. However, there was not a significant difference in the lOfc activations related to instructions between the groups, possible due to too low power. As a general comment it should be noted that the paradigms discussed above do not always show increased activation in lOfc, an effect that may be due to large susceptibility artefacts in this region.

In contrast to the fMRI analysis based purely on differences in activations between conditions, the psychophysiological interaction (PPI) analysis revealed increased functional connectivity in instructed trials (as compared to non-instructed trials) specifically for *hDP* individuals between the right lOfc and functionally defined primary nociceptive input region (right posterior insula). This result supports previous findings of an association between sensory processing and lOfc activity during an expectation modulated condition in schizophrenia (Schmack et al., 2017) and delusion-proneness (Schmack et al., 2013; Figure 4C). Interestingly, as in the study by Schmack and colleagues on delusion-proneness (Schmack et al., 2013) this functional connectivity was related to the conviction scores for the delusion-prone group (Figure 5). Although this effect was also observed for the total PDI-scores in our sample, it remained significant when tested for the normalised convictions scores. Thus, the conviction scores had a specific effect on the connectivity between lOfc and right posterior insula independent on the number of endorsed delusional items.

The PPI-analysis of the effects of instruction on fear processing also showed a significantly larger connectivity between the lOfc and the caudal anterior cingulate cortex (cACC), overlapping with fear related activation, in *hDP* compared to *lDP* (Figure 4D).

The significant group difference in lOfc functional connectivity—combined with no difference between the groups in the activation level related to fear processing—suggests mainly a difference in the re-appraisal effect between delusion-prone and control subjects. A similar region in lOfc links expectations to visual input (Bar, 2003) and mediates belief congruent information to visual processing of the random dot kinetogram illusion related to delusion-proneness (Schmack et al., 2013). Prefrontal networks, that include lOfc, are also involved in self-referential experience of presented generic stimuli in delusional patients with Schizophrenia (Lariviere et al., 2017). Based on these previous studies as well as our results, we argue that lOfc may be important for construction of higher-order priors used more readily in delusion-proneness, especially in emotional and visual processes

In a previous study on the impact of instructions on classical fear learning (Atlas et al., 2016), an effect of instructions was observed in the dorsolateral prefrontal cortex (dlPFC), stretching towards ventrolateral PFC. Our main activation in the lOfc extends towards the same area. Finally, only the delusion-prone group showed activation in the ventromedial prefrontal cortex (vmPFC) in main effect of instructions—a region previously implicated in mediation of cognitive reappraisal (Wager et al., 2008).

Cognitive neuroscience research on psychosis has recently focused on the involvement of expectations (or priors) in underlying mechanisms (Fletcher and Frith, 2009; Adams et al., 2013; Sterzer et al., 2018) and suggested that the balance between bottom-up signals and top-down influence of expectations is altered in psychotic states due to aberrant (or hyper) salience of incoming information (Kapur, 2003)—possibly linked to a hypersensitive dopamine system (Kuepper et al., 2012)—and weakened or imprecise low-level priors. Recently, hierarchical Bayesian models (Friston, 2005) have been successfully applied to explain hallucinations and underlying processes observed in psychosis-associated states (Powers et al., 2017). However, predictive coding models have so far not been able to account for both chaotic perceptions (involving imprecise priors) and delusions (involving overly precise priors). From a predictive coding perspective, the present study together with previous findings (Schmack et al., 2013; Teufel et al., 2015) suggest that individuals in psychosis-related states, including healthy delusion-prone subjects, are more prone to integrate and use higher-order beliefs (or models/priors) of the world in order to better comprehend a noisy perceptual environment. Altogether, our study and previous work on fear processing in psychosis-related states, suggest the coexistence of a weak low-level and strong high-level fear learning in psychosis-related endophenotypes.

## Data Availability Statement

The raw data supporting the conclusions of this article will be made available by the authors, without undue reservation.

## Ethics Statement

The studies involving human participants were reviewed and approved by Regional Ethical Board of Stockholm (www.epn.se). The participants provided their written informed consent to participate in this study.

## Author Contributions

PP, MI, and ALo designed the study. PP and ALo performed the experiments. All authors performed parts of the analyses, contributed to the writing, read the manuscript, and approved the submitted version.

## Conflict of Interest

The authors declare that the research was conducted in the absence of any commercial or financial relationships that could be construed as a potential conflict of interest.

## Publisher’s Note

All claims expressed in this article are solely those of the authors and do not necessarily represent those of their affiliated organizations, or those of the publisher, the editors and the reviewers. Any product that may be evaluated in this article, or claim that may be made by its manufacturer, is not guaranteed or endorsed by the publisher.

## Footnotes

1
www.epn.se

2
www.biopac.com

3 www.neurobs.com, version 9.13

4
http://www.fil.ion.ucl.ac.uk/spm
