# Supplementary material for: Enhanced Instructed Fear Learning in Delusion-Proneness
Source: Front Psychol. 2022 Apr 13;13:786778. doi: 10.3389/fpsyg.2022.786778 (PMC9043131; doi:10.3389/fpsyg.2022.786778)
Supplement: Supplementary file 1 [file Data_Sheet_1.docx]

**SUPPLEMENTARY MATERIAL**

**Enhanced instructed fear learning in delusion-proneness**

**Supplementary Results**

**General fear learning in the two groups**

In both groups the *affective learning index* increased significantly after instructions (T1 vs T0) for the instructed stimuli (*lDP*: mean=72.52, SD=74.59, paired t-test t=1.963; df=22, p=0.032 one-tailed; *hDP* mean=114.75, SD=93.26, paired t-test t=2.350, df=19, p=0.015 one-tailed). The results suggest an instructed fear learning effect for the instructed stimuli.

*Affective learning index* for the non-instructed CS pair increased significantly after acquisition (T2 vs T1) in lDP (mean T1=-0.261, SD=38.46; mean T2=63.00, SD=62.16; paired t-test t=4.405, df=22, p<0.001 one-tailed) and in hDP (mean T1=5.70, SD=47.92; mean T2 =89.45, SD=81.52; paired t-test t=6.165, df=19, p<0.001 one-tailed). The results suggest an classical fear conditioning effect for the non-instructed stimuli.

A trend towards a conditioning effect (T2 vs T1) based on *affective learning* *index* was also observed for the instructed CS pair both in lDP (mean before acquisition=72.52, SD=74.59; mean after acquisition=79.73, SD=67.93; paired t-test t=1.679, df=22, p=0.054 one-tailed) and hDP (mean before acquisition=114.75, SD=93.26; mean after acquisition=131.15, SD=100.35 – paired t-test t=1.704, df=19, p=0.053 one-tailed). The results suggest an classical fear conditioning threshold effect for the instructed stimuli. Notably, these effects are additive to the learning effects of instructions, and thereby possibly affected by ceiling effects.

**Supplementary Tables**

**Supplementary Table 1.** **Effect of Conditioning.** The main effect of the conditioning task *(****CS+ > CS−****)* led to activations in brain areas that are consistently reported in fear conditioning studies (*22*). Insula and cACC ROIs defined from the main effect of conditioning (Table 1A) were used as small volume correction (SVC) for analysis of possible group difference (Table 1D). The map was thresholded at p < 0.001 (uncorrected), k > 20 transformed voxels. ***hDP***: high delusion-proneness group. ***lDP***: low delusion-proneness group. The shown *p-values were corrected for full-brain volume (FWE-correction). Peak-activations used for the subsequent ROI-analyses (6mm sphere - including PPI-analysis) are shown in bold. cACC = caudal anterior cingulate cortex. dlPFC = dorsolateral prefrontal cortex.

| **A - Main effect of conditioning - delusion-prone and control group** | | | | | |
| --- | --- | --- | --- | --- | --- |
| ***[(iCS+ vs iCS-) + (niCS+ vs niCS-)]hDP + [(iCS+ vs iCS-) + (niCS+ vs niCS-)]lDP*** | | | | | |
|  | **Cluster level** | | **Peak level** | | |
|  | **p-value*** | **number of voxels** | **[X Y Z]** | **Z-value** | **p-value*** |
| cACC | 0.000 | 860 | **[6 8 49]** | **5.23** | **0.002** |
|  |  |  | [6 11 37] | 5.01 | 0.005 |
|  |  |  | [18 -1 70] | 4.61 | 0.024 |
| Right Insula | 0.000 | 420 | **[33 29 1]** | **5.65** | **0.000** |
|  |  |  | [54 5 7] | 4.35 | 0.066 |
| Left Insula | 0.000 | 370 | **[-30 26 1]** | **5.85** | **0.000** |
|  |  |  | [-51 2 7] | 5.05 | 0.004 |
|  |  |  | [-39 5 -2] | 3.56 | 0.609 |
| Brainstem | 0.003 | 261 | [-3 -25 -2] | 5.01 | 0.004 |
|  |  |  | [6 -19 -5] | 4.58 | 0.027 |
|  |  |  | [15 -13 4] | 4.35 | 0.066 |
| Premotor/dlPFC | 0.010 | 193 | [45 -1 46] | 5.10 | 0.003 |
| Right temporoparietal junction | 0.012 | 184 | [66 -34 16] | 4.77 | 0.013 |
|  |  |  | [48 -22 22] | 4.05 | 0.182 |
| **B - Main effect of conditioning – delusion-prone group** | | | | | |
| ***[(iCS+ vs iCS-) + (niCS+ vs niCS-)]hDP*** | | | | | |
|  | **Cluster level** | | **Peak level** | | |
|  | **p-value*** | **number of voxels** | **[X Y Z]** | **Z-value** | **p-value*** |
| cACC | 0.023 | 168 | [9 8 49] | 3.61 | 0.568 |
|  |  |  | [-6 8 40] | 3.47 | 0.703 |
|  |  |  | (9 14 37] | 3.36 | 0.805 |
| Premotor/dlPFC | 0.254 | 56 | [42 -1 46] | 3.81 | 0.370 |
| Right Insula | 0.492 | 29 | [33 29 1] | 3.47 | 0.705 |
|  |  |  | [39 20 7] | 3.47 | 0.810 |
|  |  |  |  |  |  |
|  |  |  |  |  |  |
| **C - Main effect of conditioning control group** | | | | | |
| ***[(iCS+ vs iCS-) + (niCS+ vs niCS-)]lDP*** | | | | | |
|  | **Cluster level** | | **Peak level** | | |
|  | **p-value*** | **number of voxels** | **[X Y Z]** | **Z-value** | **p-value*** |
| Left insula | 0.000 | 315 | [-30 23 4] | 6.20 | 0.000 |
|  |  |  | [-45 5 4] | 4.76 | 0.029 |
| cACC | 0.000 | 292 | [0 8 49] | 4.16 | 0.234 |
|  |  |  | [6 8 34] | 3.89 | 0.459 |
|  |  |  | [3 2 40] | 3.72 | 0.648 |
| Right insula | 0.001 | 235 | [30 26 -8] | 4.53 | 0.069 |
|  |  |  | [30 32 7] | 4.53 | 0.069 |
|  |  |  | [40 11 7] | 3.40 | 0.916 |
| Brainstem | 0.002 | 191 | [3 -16 1] | 4.76 | 0.029 |
|  |  |  | [6 -31 -8] | 3.74 | 0.618 |
|  |  |  |  |  |  |
|  |  |  |  |  |  |
| **D - Main effect of conditioning - group difference - Small Volume Correction (ROI)** | | | | | |
| ***[(iCS+ vs iCS-) + (niCS+ vs niCS-)]lDP - [(iCS+ vs iCS-) + (niCS+ vs niCS-)]hDP*** | | | | | |
| ***[(iCS+ vs iCS-) + (niCS+ vs niCS-)]hDP - [(iCS+ vs iCS-) + (niCS+ vs niCS-)]lDP*** | | | | | |
| ns | | | | | |
|  |  |  |  |  |  |
|  |  |  |  |  |  |
| **E - Main effect of conditioning - group difference – whole brain** | | | | | |
| ***[(iCS+ vs iCS-) + (niCS+ vs niCS-)]lDP - [(iCS+ vs iCS-) + (niCS+ vs niCS-)]hDP*** | | | | | |
| ***[(iCS+ vs iCS-) + (niCS+ vs niCS-)]hDP - [(iCS+ vs iCS-) + (niCS+ vs niCS-)]lDP*** | | | | | |
| ns | | | | | |

**Supplementary Table 2.** **Effect of Instructions ROI analysis (A-D) and whole-brain analysis (E-H).** The main effect of instructions resulted in the activation of brain regions involved in reappraisal conditions, i.e. lOfc bilaterally. The small volume correction analyses (A-D) were performed using an anatomically defined bilateral lOfc ROI. The effects of learning did not yield any significant or non-significant activation in the ***lDP*** (C), while the effects survived full-brain correction in the ***hDP*** (F). The general activation pattern (A and D) seems to be mainly driven by individuals in the ***hDP***. The map was thresholded at p < 0.001 (uncorrected), k > 20 transformed voxels. ***hDP***: high delusion-proneness group. ***lDP***: low delusion-proneness group. The shown *p-values were corrected for full brain volume (FWE-correction). SVC = small volume correction.

| **A - Effect of Instructions – delusion-prone and control group - SVC anatomically defined ROI bilateral lOfc** | | | | | |
| --- | --- | --- | --- | --- | --- |
| ***[(iCS+ + iCS-) vs (niCS+ + niCS-)]hDP + [(iCS+ + iCS-) vs (niCS+ + niCS-)]lDP*** | | | | | |
|  | **Cluster level** | | **Peak level** | | |
|  | **p-value*** | **number of voxels** | **[X Y Z]** | **Z-value** | **p-value*** |
| Left lOfc | 0.001 | 155 | [-45 32 -5] | 4.63 | 0.001 |
|  |  |  | [-30 23 -14] | 4.22 | 0.004 |
|  |  |  | [-51 17 -5] | 3.77 | 0.056 |
| Right lOfc | 0.006 | 77 | [48 29 -8] | 4.53 | 0.001 |
|  |  |  | [45 38 -11] | 4.15 | 0.006 |
|  |  |  |  |  |  |
|  |  |  |  |  |  |
| **B - Effect of Instructions – delusion-prone group - SVC anatomically defined ROI bilateral lOfc** | | | | | |
| ***[(iCS+ + iCS-) vs (niCS+ + niCS-)]hDP*** | | | | | |
|  | **Cluster level** | | **Peak level** | | |
|  | **p-value*** | **number of voxels** | **[X Y Z]** | **Z-value** | **p-value*** |
| Left lOfc | 0.002 | 92 | [-30 23 -14] | 4.1 | 0.009 |
|  |  |  | [-39 17 -17] | 3.73 | 0.032 |
|  |  |  | [-45 32 -5] | 3.51 | 0.062 |
|  |  |  | [-33 35 -5] | 3.33 | 0.101 |
| Right lOfc | 0.002 | 86 | [51 26 -8] | 4 | 0.014 |
|  |  |  | [42 32 -11] | 3.87 | 0.021 |
|  |  |  | [51 38 -11] | 3.5 | 0.064 |
| **C - Effect of Instructions - control group - SVC anatomically defined ROI bilateral lOfc** | | | | | |
| ***[(iCS+ + iCS-) vs (niCS+ +niCS-)]lDP*** | | | | | |
| ns | | | | | |
|  |  |  |  |  |  |
|  |  |  |  |  |  |
| **D - Effect of Instructions - group difference - SVC anatomically defined ROI bilateral lOfc** | | | | | |
| ***[(iCS+ + iCS-) vs (niCS+ + niCS-)]lDP - [(iCS+ + iCS-) vs (niCS+ + niCS-)]hDP*** | | | | | |
| ***[(iCS+ + iCS-) vs (niCS+ + niCS-)]hDP - [(iCS+ + iCS-) vs (niCS+ + niCS-)]lDP*** | | | | | |
| ns | | | | | |
| **E - Effect of Instructions - delusion-prone and control group – full-brain correction** | | | | | |
| ***[(iCS+ + iCS-) vs (niCS+ + niCS-)]hDP + [(iCS+ +iCS-) vs (niCS+ + niCS-)]lDP*** | | | | | |
|  | **Cluster level** | | **Peak level** | | |
|  | **p-value*** | **number of voxels** | **[X Y Z]** | **Z-value** | **p-value*** |
| Left lOfc | 0 | 428 | [-45 32 -5] | 4.63 | 0.025 |
|  |  |  | [-30 23 -14] | 4.22 | 0.113 |
|  |  |  | [-51 14 7] | 3.88 | 0.325 |
| Right lOfc | 0.032 | 128 | [48 29 -8] | 4.53 | 0.036 |
|  |  |  | [45 38 -11] | 4.15 | 0.145 |
| Premotor/dlPFC | 0.009 | 182 | [48 -1 52] | 4.38 | 0.065 |
|  |  |  | [39 -4 61] | 3.84 | 0.351 |
|  |  |  | [33 -16 22] | 3.61 | 0.594 |
|  |  |  |  |  |  |
|  |  |  |  |  |  |
|  |  |  |  |  |  |
| **F - Effect of Instructions - delusion-prone group – full-brain correction** | | | | | |
| ***[(iCS+ + iCS-) vs (niCS+ + niCS-)]hDP*** | | | | | |
|  | **Cluster level** | | **Peak level** | | |
|  | **p-value*** | **number of voxels** | **[X Y Z]** | **Z-value** | **p-value*** |
| Left lOfc | 0.006 | 156 | [-30 23 -14] | 4.1 | 0.262 |
|  |  |  | [-39 17 -20] | 3.76 | 0.585 |
|  |  |  | [-30 35 -2] | 3.54 | 0.805 |
| Right lOfc | 0.03 | 105 | [51 26 -8] | 4 | 0.349 |
|  |  |  | [42 32 -11] | 3.87 | 0.471 |
|  |  |  | [51 38 -11] | 3.5 | 0.838 |
| vmPFC | 0.009 | 145 | [0 50 40] | 3.62 | 0.733 |
|  |  |  | [6 59 16] | 3.67 | 0.783 |
|  |  |  | [12 53 40] | 3.5 | 0.84 |
|  |  |  |  |  |  |
|  |  |  |  |  |  |
| **G - Effect of Instructions - control group – full-brain correction** | | | | | |
| ***[(iCS+ + iCS-) vs (niCS+ +niCS-)]lDP*** | | | | | |
| Ns | | | | | |
|  |  |  |  |  |  |
|  |  |  |  |  |  |
| **H - Effect of Instructions - group difference – full-brain correction** | | | | | |
| ***[(iCS+ + iCS-) vs (niCS+ + niCS-)]lDP - [(iCS+ + iCS-) vs (niCS+ + niCS-)]hDP*** | | | | | |
| ***[(iCS+ + iCS-) vs (niCS+ + niCS-)]hDP - [(iCS+ + iCS-) vs (niCS+ + niCS-)]lDP*** | | | | | |
| Ns | | | | | |

**Supplementary Table 3.** **Instructed conditioning - full brain analysis.**  Instructed conditioning resulted in the activation of brain regions similar to the ones activated in the main effect of general conditioning. Insula and cACC ROIs defined from the main effect of conditioning (Table 1A) were used as small volume correction (SVC) for analysis of possible group difference (Table 3D). The map was thresholded at p < 0.001 (uncorrected), k > 20 transformed voxels. ***hDP***: high delusion-proneness group. ***lDP***: low delusion-proneness group. The shown *p-values were corrected for full brain volume (FWE-correction). cACC = caudal anterior cingulate cortex. dlPFC = dorsolateral prefrontal cortex.

| **A - Effect of conditioning - Instructed - delusion-prone and control group** | | | | | |
| --- | --- | --- | --- | --- | --- |
| ***(iCS+ vs iCS-)hDP + (iCS+ vs iCS-)lDP*** | | | | | |
|  | **Cluster level** | | **Peak level** | | |
|  | **p-value** | **number of voxels** | **[X Y Z]** | **Z-value** | **p-value** |
| Right insula | 0.000 | 428 | [33 29 4] | 5.95 | 0.000 |
|  |  |  | [54 5 7] | 4.23 | 0.990 |
|  |  |  | [45 8 7] | 4.07 | 0.166 |
| Left insula | 0.001 | 313 | [-30 26 4] | 5.36 | 0.001 |
|  |  |  | [-51 -1 7] | 4.65 | 0.020 |
|  |  |  | [-63 5 25] | 3.76 | 0.395 |
| Brainstem | 0.015 | 176 | [3 -16 -8] | 4.76 | 0.013 |
|  |  |  | [-6 -22 1] | 4.18 | 0.115 |
|  |  |  | [15 -13 4] | 4.15 | 0.126 |
| cACC | 0.000 | 510 | [9 11 37] | 4.69 | 0.017 |
|  |  |  | [-9 -1 49] | 4.54 | 0.031 |
|  |  |  | [-9 8 40] | 4.35 | 0.062 |
| Right Premotor/dlPFC | 0.044 | 126 | [42 -1 43] | 4.60 | 0.025 |
|  |  |  | [45 2 55] | 4.11 | 0.146 |
| Right temporoparietal junction | 0.016 | 173 | [48 -25 22] | 4.50 | 0.037 |
|  |  |  | [54 -37 19] | 4.17 | 0.119 |
|  |  |  | [66 -34 16] | 4.09 | 0.155 |
| Left temporoparietal junction | 0.036 | 135 | [-54 -28 19] | 4.42 | 0.049 |
| **B - Effect of conditioning - Instructed - delusion-prone group** | | | | | |
| ***(iCS+ vs iCS-)hDP*** | | | | | |
|  | **Cluster level** | | **Peak level** | | |
|  | **p-value** | **number of voxels** | **[X Y Z]** | **Z-value** | **p-value** |
| Right insula | 0.393 | 38 | [33 29 4] | 3.77 | 0.413 |
|  |  |  | [36 14 7] | 3.11 | 0.951 |
| Right premotor | 0.323 | 46 | [42 -1 43] | 3.57 | 0.607 |
|  |  |  |  |  |  |
|  |  |  |  |  |  |
| **C - Effect of conditioning - Instructed - control group** | | | | | |
| ***(iCS+ vs iCS-)lDP*** | | | | | |
|  | **Cluster level** | | **Peak level** | | |
|  | **p-value** | **number of voxels** | **[X Y Z]** | **Z-value** | **p-value** |
| Right insula | 0.001 | 220 | [30 26 -2] | 4.91 | 0.016 |
| Left insula | 0.001 | 212 | [-30 23 4] | 4.76 | 0.030 |
|  |  |  | [-51 2 7] | 3.93 | 0.431 |
| Brainstem | 0.008 | 136 | [6 -16 -2] | 4.43 | 0.100 |
|  |  |  | [-3 -16 -2] | 4.13 | 0.256 |
| cACC | 0.000 | 448 | [9 -1 49] | 3.74 | 0.634 |
|  |  |  | [9 -1 37] | 3.73 | 0.639 |
|  |  |  | [9 8 37] | 3.58 | 0.787 |
|  |  |  |  |  |  |
|  |  |  |  |  |  |
| **D - Effect of conditioning - Instructed - group difference - Small Volume Correction** | | | | | |
| ***(iCS+ vs iCS-)lDP - (iCS+ vs iCS-)hDP*** | | | | | |
| ***(iCS+ vs iCS-)hDP - (iCS+ vs iCS-)lDP*** | | | | | |
| ns | | | | | |
| **E – Effect of conditioning - Instructed - group difference – full-brain correction** | | | | | |
| ***(iCS+ vs iCS-)lDP - (iCS+ vs iCS-)hDP*** | | | | | |
| ***(iCS+ vs iCS-)hDP - (iCS+ vs iCS-)lDP*** | | | | | |
| ns | | | | | |

**Supplementary Table 4.** **Non-Instructed conditioning - full brain analysis.** Non-instructed conditioning resulted in the activation of brain regions similar to the ones activated in the main effect of general conditioning. Insula and cACC ROIs defined from the main effect of conditioning (Table 1A) were used as small volume correction (SVC) for analysis of possible group difference (Table 4E). The map was thresholded at p < 0.001 (uncorrected), k > 20 transformed voxels. ***hDP***: high delusion-proneness group. ***lDP***: low delusion-proneness group. The shown *p-values were corrected for full brain volume (FWE-correction). cACC = caudal anterior cingulate cortex.

| **A - Effect of conditioning - Non-Instructed - delusion-prone and control group** | | | | | |
| --- | --- | --- | --- | --- | --- |
| ***(iCS+ vs iCS-)hDP + (iCS+ vs iCS-)lDP*** | | | | | |
|  | **Cluster level** | | **Peak level** | | |
|  | **p-value** | **number of voxels** | **[X Y Z]** | **Z-value** | **p-value** |
| Left insula | 0.058 | 123 | [-30 26 1] | 4.95 | 0.005 |
| cACC | 0.011 | 210 | [9 5 58] | 3.62 | 0.499 |
|  |  |  | [-9 11 40] | 3.62 | 0.504 |
|  |  |  | [6 14 37] | 3.49 | 0.637 |
| Brainstem | 0.086 | 104 | [9 -22 -5] | 4.06 | 0.125 |
|  |  |  | [6 -22 -2] | 3.28 | 0.153 |
| Right insula | 0.177 | 71 | [33 29 1] | 4.17 | 0.110 |
|  |  |  |  |  |  |
|  |  |  |  |  |  |
| **B - Effect of conditioning - Non-Instructed - delusion-prone group** | | | | | |
| ***(iCS+ vs iCS-)hDP*** | | | | | |
| ns | | | | | |
|  |  |  |  |  |  |
| **C - Effect of conditioning - Non-Instructed - control group** | | | | | |
| ***(iCS+ vs iCS-)hDP*** | | | | | |
|  | **Cluster level** | | **Peak level** | | |
|  | **p-value** | **number of voxels** | **[X Y Z]** | **Z-value** | **p-value** |
| Left insula | 0.112 | 72 | [-30 26 4] | 4.41 | 0.085 |
|  |  |  |  |  |  |
|  |  |  |  |  |  |
| **D - Effects of Conditioning - Non-instructed - group difference** | | | | | |
| ***(niCS+ vs niCS-)lDP - (niCS+ vs niCS-)hDP*** | | | | | |
| ***(niCS+ vs niCS-)hDP - (niCS+ vs niCS-)lDP*** | | | | | |
| ns | | | | | |

| **E - Effects of Conditioning - Non-instructed - group difference - Small Volume Correction** |
| --- |
| ***(niCS+ vs niCS-)lDP - (niCS+ vs niCS-)hDP*** |
| ***(niCS+ vs niCS-)hDP - (niCS+ vs niCS-)lDP*** |
| ns |

**Supplementary Table 5.** **Effect of pain - full brain analysis.**

The main effect of pain resulted in the activation of brain regions usually found in the pain network: caudal ACC (cACC), bilateral mid- and posterior insula. We only report maximally activated voxels in cACC and posterior insula bilaterally. As there was no proper control condition for the pain, the activation pattern observed in this contrast was wider than the one generally observed in pain studies using a low-level control. The map was thresholded at p < 0.001 (uncorrected), k > 20 transformed voxels. The shown *p-values were corrected for full brain volume (FWE-correction). Peak-activations used for the subsequent PPI-analyses are shown in bold.

| **Effect of pain - delusion-prone and control group** | | | |
| --- | --- | --- | --- |
|  |  |  |  |
|  | **Peak level** | | |
|  | **Z-values** | **[X Y Z]** | **p-value*** |
| cACC | Inf | [-3 2 37] | <0.0001 |
| *Right Insula* |  |  |  |
| Posterior upper Insula | Inf | **[36 -16 4]** | **<0.0001** |
| Posterior lower Insula | Inf | [39 -13 -8] | <0.0001 |
| Mid Insula | Inf | [42 11 -5] | <0.0001 |
| *Left Insula* |  |  |  |
| Mid Insula | Inf | [-39 7 -5] | <0.0001 |
| Posterior lower Insula | Inf | [-39 -10 -2] | <0.0001 |
| Posterior upper Insula | Inf | [-39 -16 13] | <0.0001 |
